# Correlation of Geographic Variables with the Incidence Rate of Dengue Fever in Mexico: A 38-Year Study

**DOI:** 10.3390/microorganisms12122661

**Published:** 2024-12-22

**Authors:** Porfirio Felipe Hernández Bautista, David Alejandro Cabrera Gaytán, Alfonso Vallejos Parás, Olga María Alejo Martínez, Lumumba Arriaga Nieto, Brenda Leticia Rocha Reyes, Carmen Alicia Ruíz Valdez, Leticia Jaimes Betancourt, Gabriel Valle Alvarado, Yadira Pérez Andrade, Alejandro Moctezuma Paz

**Affiliations:** 1 Coordination of Quality of Supplies and Specialized Laboratories, Mexican Institute of Social Security, Mexico City 07760, Mexico; porfirio.hernandez@imss.gob.mx; 2 Coordination of Epidemiological Surveillance, Mexican Institute of Social Security, Mexico City 03100, Mexico; alfonso.vallejos@imss.gob.mx (A.V.P.); lumumba.arriaga@imss.gob.mx (L.A.N.); gabriel.valle@imss.gob.mx (G.V.A.); yadira.perezan@imss.gob.mx (Y.P.A.); 3 General Zone Hospital with Family Medicine No. 28-IMSS-Costa Rica, Culiacán 80430, Mexico; olga.alejo@imss.gob.mx; 4 High Specialty Medical Unit 08, Specialty Hospital, Ciudad Obregón 85120, Mexico; brenda.rocha@imss.gob.mx; 5 Regional General Hospital No. 1, Ciudad Obregón 85110, Mexico; carmen.ruizva@imss.gob.mx; 6 Family Medicine Unit No. 7, Mexican Institute of Social Security, Mexico City 14370, Mexico; leticia.jaimesb@imss.gob.mx; 7 Coordination of Health Research, Mexican Institute of Social Security, Mexico City 06720, Mexico; alejandro.moctezuma@imss.gob.mx

**Keywords:** dengue, epidemiological surveillance, precipitation, temperature

## Abstract

Background: Dengue is a viral disease transmitted by the mosquitoes *Aedes*, which is characterized by fever, myalgia and arthralgia. In some cases, it can be fatal. For many years, dengue fever has been endemic to Mexico; however, few studies have investigated the historical and current extents of dengue fever at the national level or considered the effects of variables such as temperature, precipitation and elevation on its occurrence. Methods: An ecological study was carried out to compare the incidence rates of different types of dengue fever per hundred thousand inhabitants with temperature, precipitation and elevation between 1985 and 2023 in Mexico. The sources of information were the public records of the Ministry of Health and the National Meteorological Service. Multiple linear regression analysis was performed with Pearson and Spearman correlation coefficients at an alpha of <0.05. Results: The global linear regression presented an R^2^ of 0.68 between the mean temperature and the cases of haemorrhagic dengue/severe/with warning signs. The degree of rainfall was not strongly correlated with the incidence rate, except in the eastern part of the country, where average temperature was also strongly correlated with the incidence rate. Nonsevere/classic dengue was most common from 1501 to 2000 m elevation, whereas severe forms of the disease were more prevalent at elevations greater than 2000 m.

## 1. Introduction

Dengue fever is a disease caused by a virus that is transmitted by mosquitoes of the genus *Aedes*, mainly in countries in the tropics and subtropics; which is characterized by fever, myalgia and arthralgia, which in some cases can be fatal [1,2]. The dengue virus belongs to *Orthornaviridae*, the family *Flaviviridae* and the genus *Flaviviridae*. It is a positive-stranded single-stranded RNA virus and consists of four serotypes DENV 1–4. Due to its mode of transmission, it has also been classified as an arbovirus, i.e., related to arthropods. In this case, transmission occurs mainly through the species *A. aegypti* and *A. albopictus* [1,2]. Dengue is considered endemic to Mexico, with residents being prone to transmission [1,2]. Four serotypes (DENV 1, 2, 3, 4) are found in Mexico, and in some states, there is evidence of the simultaneous circulation of more than one serotype [3,4,5].

Tropical diseases are diseases whose incidence increases in tropical climates. This occurs due to the life cycles of the vectors, reservoirs and hosts of these diseases, which are directly influenced by climatic variables and the dynamics of ecosystems [6]. Currently, due to global warming, the climate is changing, which favours the growth of vectors [7]. Various studies have aimed to understand the impact of climate change on health at the individual and population levels [8] since there is a positive relationship between rainfall, humidity and wind conditions and the incidence of vector-borne diseases, including dengue [9,10].

In Mexico, studies have shown that climate change increases the incidence of dengue [11,12]. Additionally, a spatiotemporal study with data from 20 years (1995–2015), carried out in Mexico, highlighted that the greatest relative risks for suffering from dengue fever were in the states located on coasts from the Pacific and the Gulf of Mexico, where the climate is tropical [13].

Therefore, the correlations between environmental temperature, elevation and rainfall were evaluated with the incidence rate of dengue fever in the states of the Mexican Republic over five years. The hypothesis we tested is that there is a correlation between temperature and rainfall and the incidence rate of dengue fever in Mexico.

## 2. Materials and Methods

### 2.1. General Description of the Study

This was a national-based retrolective ecological study. The incidence rates of dengue per 100,000 inhabitants were obtained from the General Directorate of Epidemiology [3], whereas the temperature, rainfall and elevation records were obtained from the National Meteorological Service (NMS) [14]. In both cases, the study period was between 1985 and 2023. An adjustment was made for the different types of dengue fever. Classic dengue and haemorrhagic dengue were more common between 1985 and 2016, while from 2017 to 2023, non-severe dengue, dengue with warning signs and severe dengue were common; therefore, the cases were grouped as follows: (1) nonsevere dengue and classic dengue and (2) haemorrhagic dengue together with dengue with warning signs and severe dengue. The states of the Republic were grouped by geographical regions: centre-north (Aguascalientes, Guanajuato, Querétaro, San Luis Potosí and Zacatecas), centre-south (Mexico City, State of Mexico and Morelos), northeast (Coahuila, Nuevo León and Tamaulipas), northwest (Baja California, Baja California Sur, Chihuahua, Durango, Sinaloa and Sonora), west (Colima, Jalisco, Michoacán and Nayarit), east (Hidalgo, Puebla, Tlaxcala and Veracruz), southeast (Campeche, Quintana Roo, Tabasco and Yucatán) and southwest (Chiapas, Guerrero and Tabasco). Owing to the annual climatic variability, as well as the presentation of cases in epidemic outbreaks, it was decided to use the five-year period (Q) as a unit of comparison; a total of seven five-year periods and a group with three years were obtained. The maximum height of each state was classified into the following groups: <500, 501 to 1500, 1501 to 2000 and >2000 m above sea level. The median temperatures in degrees centigrade, rainfall (mm) and incidence rates were used. The maximum elevation of each of the states was considered for the final analysis. No minimum sample size calculation was performed because all the records of the study period were used. Since the data are open access, they do not require evaluation by a research committee.

### 2.2. Statistical Analysis

A temporal trend analysis was performed, and the data were broken down by state; thus, multiple linear regression with Pearson and Spearman’s correlation coefficients at an alpha of <0.05 was performed. We used Epiinfo CDC v. 7.2.5.0 and Software R v. 4.2.5.0. The variables were adjusted according to the collinearity of the significant variables.

## 3. Results

**Figure 1:**
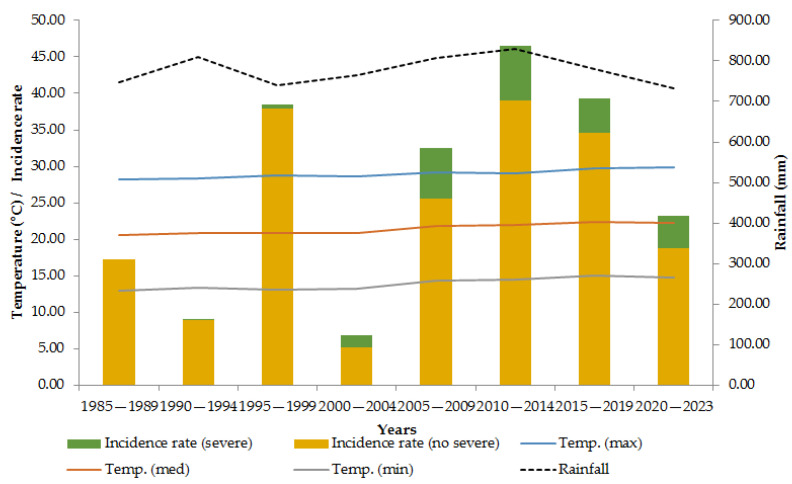
Dengue incidence rate by type and temperature in Mexico, 1985—2023.

This study revealed an average temperature increase in Mexico of 1.6 degrees Celsius, although this was not regionally homogeneous (Table S1). When grouped by five-year periods, there were three periods with a relatively high incidence rate, and there was a difference of 0.3 °C in the maximum temperature and a difference of 1.6 °C between them; this trend was not observed for rainfall, except for the period 2010–2014 (Figure 1 and Table S2).

The global linear regression had an R^2^ of 0.68 for the relationship of the mean temperature with the cases of haemorrhagic dengue/severe/with warning signs. The Pearson statistic was 0.83 (*p* = 0.0115) (Table 1). The Spearman test result was 0.7892 (*p* = 0.0199). However, when rainfall was included in the model, R^2^ = 0.81 and *p* = 0.0152 were obtained for the same variables.

When performing the linear regression between temperature and the rate of classic/nonsevere dengue, by region, an R^2^ of 0.88, an F of 43, and a *p* = 0.0006 were observed in the eastern region due to the minimum temperature, whereas an increase in temperature and dengue haemorrhagic fever/severe/with warning signs (R^2^ = 0.91, *p* = 0.0002), was found for the mean temperature (Table 2). When rain was added to the model, R^2^ = 0.92, *p* = 0.0019 (Table 3) was observed.

When analysed by elevation, for areas between 1501 and 2000 metres above sea level, there was a positive correlation between the minimum temperature and the incidence rate of classic/nonsevere dengue (R^2^ = 0.82, *p* = 0.0019; Pearson = 0.9058, *p* = 0.0019; Spearman = 8571, *p* = 0.0065). For dengue haemorrhagic/severe/with warning signs, (R^2^ = 0.84, *p* = 0.0015; Pearson = 0.9140, *p* = 0.0015; Spearman = 7638 *p* = 0.0274) at a height greater than 2000 metres (Table 4, Tables S3 and S4).

The degree of rainfall was not strongly correlated with the incidence rate, with the exception of the eastern part of the country and the average maximum temperature in most areas (Table 5). However, when rain was added to the model, the R^2^ rose to 0.90 (*p* = 0.0029) for the same elevation (Table 6).

When analysed by height, for the area between 1501 and 2000 m above sea level, there was a correlation between the increase in minimum temperature and classic dengue (R^2^ = 0.82, *p* = 0.0019, Pearson = 0.9058, *p* = 0.0019; Spearman = 8571, *p* = 0.0065). For haemorrhagic dengue, R^2^ = 0.84, *p* = 0.0015, Pearson = 0.9140, *p* = 0.0015; Spearman = 7638, *p* = 0.0274 at an elevation greater than 2000 metres. When rain was added to the model, R^2^ increased to 0.90 (*p* = 0.0029) for the same height.

**Figure 2:**
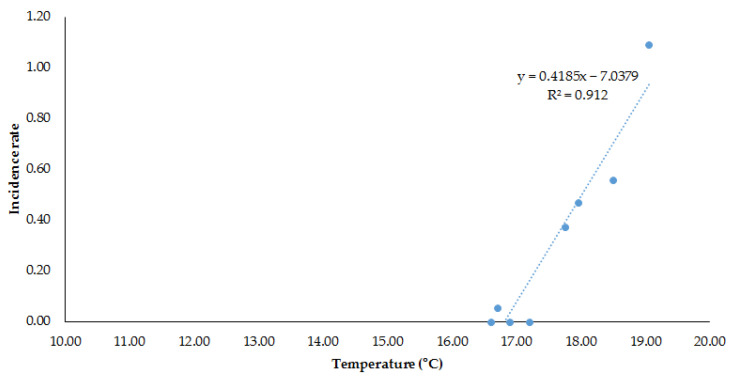
Haemorrhagic dengue/severe dengue and average temperature in the Eastern Region of Mexico.

Finally, for the four states of the eastern zone in five-year periods, there was a strong correlation between the mean temperature and the incidence rate of severe dengue in the last 18 years (Figure 2): between 1985 and 1989, the average temperature was 16.60 °C; between 1990 and 1994, it was 16.90 °C; for 1995–1999, it was 16.70 °C; from 2000 to 2004, it was 17.20 °C; between 2005 and 2009, it rose to 17.95 °C; for 2015–2019, it was 18.50 °C; and finally, in the last three years, it was 19.05 °C, showing incidence rates were increasing from 2005.

## 4. Discussion

The purpose of this study was to explain the influence of temperature, rainfall and elevation on the incidence rate of dengue. According to the analysis, the highest correlation was with the mean and maximum temperatures, mainly in the eastern part of the country.

Climate change has caused an average global increase in temperature of 1.4 °C, according to what was observed by Mendoza-Cano et al. [15]. In our study, the average temperature increase in Mexico was 1.6 °C over 38 years.

On the other hand, the increase in maximum temperature was negatively related to the dengue fever burden [15]; however, the coefficient of determination was 5%. We observed negative correlations, but the correlations were not statistically significant for the maximum temperatures, which can be explained by the very high temperature not being very favourable for the vector, as determined by Jia P et al. [16]. The average temperature is optimal for the vector, and a very significant correlation was observed with this temperature. This difference in the results may have occurred because the data were analysed by region and height and because the precipitation variable was added to the model. The change in the analysis window to five years may have also lessened the effect of the seasonal variability of the variables.

The relation of the increase in the temperature of the temperate zones to the increase in the incidence rate of dengue can be explained by improved conditions for reproduction by members of the *Aedes* genus (i.e., the vector) [17]. This shift in conditions may favour reproduction throughout the year, which does not occur in extremely tropical climates.

Mexico has several ecosystems and microclimates [5], causing heterogeneity in the distribution of dengue fever; for example, in Veracruz (a state located on the Gulf of Mexico), it has hyperendemic conditions, where the four serotypes of dengue circulate, whereas in other states, such as Tlaxcala or Mexico City, the reported cases are imported [1,2]. However, the presence of the vector has already been documented in Mexico City and the metropolitan area of the State of Mexico [4,5].

This study revealed that an increase in temperature affects the incidence rate of dengue regionally, especially the occurrence of haemorrhagic/severe presentation/with warning signs. This finding should serve as a warning for localities where no cases have occurred, including those with an elevation that exceeds 2000 m, such as Mexico City. In the city and its metropolitan area, there are causal factors for the introduction and colonization of *Aedes aegypti*, such as continuous urbanization (legal and illegal), poor living conditions and overcrowding of homes, insufficient access to drinking water and sewerage and poor waste management, climate change and the increase in water and air temperatures in urban areas [18,19], and the mobility of individuals from places with permanent presence of the mosquito, which can induce dispersal of the vector and the virus [20,21].

In general, despite the seasonality related to the rainy season in Mexico, the pattern of dengue fever is not cyclical and depends on the prevalent serotype in circulation; in this sense, the explosiveness of epidemics by region has been a function of the susceptibility of a population with previous contact with the circulating serotype. Between 1990 and 1992, the prevalent serotypes were DENV-2 and DENV-4. In the following year, DENV-1 was introduced, with simultaneous circulation of serotypes 1, 2 and 4 between 1993 and 1995. However, between 1996 and 2000, four serotypes were present. Between 2001 and 2005, DENV-2 was predominant; between 2006 and 2011, DENV-1 was predominant; and in the following two years, DENV-2 was introduced; from 2014 to 2018, DENV-1 was predominant; from 2019 to 2022, DENV-2 was predominant; and finally, between 2023 and 2024, DENV-3 was predominant. DENV-3 stopped circulating in 2007, so the increases in the five-year period are due mainly to DENV-1, DENV-2 and, more recently, DENV-3 [3,4,22]. The rainfall fluctuated between 1985 and 1995, which was not reflected in increases in the incidence rate; nationally, there were coincidences in 1997 and 2013. Although dengue outbreaks in America have been described with a periodicity of every 3 or 5 years, as described by Martín JL et al., 2010, and Brathwaite Dick O, et al., 2012 [23,24] there is an irregular pattern in the incidence rates, which may depend on the susceptibility of the population, which is why, in this study, it was preferred to analyse every five years to reduce the variability of the reported rates. Thus, specifically, in the eastern zone, this correlation was evidenced mainly by the incidence rate of dengue with warning signs/severe/haemorrhagic, consistent with the findings of a previous study which reported that being in Veracruz (located in the eastern zone and in the Gulf of Mexico) was an important risk for the development of the disease [22]. Similar findings have been reported in other studies in Mexico [22,25]. Rain affects dengue behaviour mainly by increasing the number of vector breeding sites. When people store water in open containers during dry seasons, a negative correlation may occur. For southern-southeastern areas (Chiapas, Guerrero, Oaxaca, Campeche, Quintana Roo, Yucatán, Veracruz de Ignacio de la Llave and Tabasco), the opposite is true: these areas receive more than half of the renewable water per year (67.2%) [26]. Rainfall was associated with the incidence rate of cases, specifically at an altitude between 500 and 1500 m, which may be related to better conditions for the vector to reproduce. At other altitudes or regions, there was no major impact. However, their inhabitants have less access to water since they do not have basic services, such as plumbing, inside houses. In Mexico, the percentage of homes with plumbing was 99.6% (in 2020); the remaining homes obtained water by hauling it from an external source, for example, from the community tap, another home, a pipe, a well, or a river. In 1990, the percentage of the population with plumbing for drinking water was 77.1%; in 2000, it was 85.2%; and in 2010, it was 88.7%. The southeastern, southwestern and eastern states (specifically, Veracruz) presented the greatest lags [27]. A total of 36.3% (46.8 million people) of the population is living in poverty and lacks basic housing services, while the elimination of breeding sites and access to medical care is a challenge for 17.8% of the population (22.9 million people) [28]. In Mexico, prevention campaigns by authorities have focused on the elimination of breeding sites by keeping yards clean” or eliminating breeding sites. It should be noted that states such as Nuevo León and Morelos, despite not having a coastline, present climatic conditions in certain areas that are suitable for the development of the vector and have had previous cases of dengue fever [22,23,29]. Similarly, it has been reported that the states of Jalisco, Colima and Nayarit, located on the coast of the Pacific Ocean, presented the highest risk of having cases of classic dengue in 2009 related to rainfall, whereas for haemorrhagic dengue, the areas with the greatest risk were Campeche, Quintana Roo, Yucatán, Tabasco, Chiapas, Oaxaca and Veracruz between 2006 and 2014; this is consistent with the results for the eastern and western areas in the correlation model (Table 5). The results of this study can be used to inform budgets for the states with the highest risk and prioritize health care.

*Aedes aegypti* has been the vector for the transmission of dengue in America. In 1986, *Aedes albopictus* was introduced to Texas, USA, and later spread through the border states of Mexico [30]. It is mainly located in the Pacific of Mexico and the southeast of the country, although from 1988 to 2021, its spread throughout the country increased [31]. In 2006, it was observed that 84% of cement sinks contained *Aedes aegypti pupae* [32]. Also, in 2014, in Mérida, Yucatán, the association between the presences of adult *Aedes* with higher positivity of ovitraps was identified [33]. *Aedes aegypti* can be found from 1700 to 2130 m in a scarce form; however, with the climatic conditions of global warming, there are favourable conditions for its proliferation [34], which was demonstrated in 2015 when *Aedes* larvae were found in two locations in Mexico City located at an altitude of 2250 m [4]. Dávalos-Becerril et al. detected an increasing presence of *Aedes aegypti*, which was detected for three consecutive years (2015–2017), predominantly in the warmer microclimates of the city. We found a possible correlation between increasing temperature and *Aedes aegypti* and *Aedes albopictus* expanding range, which is consistent with our findings [5]. From 2016 to 2018, ovitraps were monitored in 15 houses in two communities in the state of Chiapas. It was observed that eggs were present in the houses 2.3 to 3.2 times more in the rainy season than in the dry season, while in the periphery of the house, there were 4.8 to 5.1 times more eggs during the season with the highest rainfall [35]. In our analysis, we found a correlation with rainfall and dengue cases in the eastern part of the country.

Finally, humidity is a key factor in the proliferation of the *Aedes aegypti* mosquito, which is responsible for transmitting diseases such as dengue. Previous studies have shown that high humidity conditions favour mosquito survival and the multiplication of larvae, which can increase the incidence of vector-borne diseases like dengue. The combination of high humidity and temperature creates an environment conducive to the mosquito’s life cycle, increasing its population in tropical and subtropical areas. This phenomenon has been documented in several studies [36] and has been observed especially in areas with high rainfall [37]. Additionally, other research has indicated that relative humidity above 60% is ideal for vector reproduction [38], while a lack of rain or drought can reduce the availability of habitats for larvae [39].

The operational definitions that were used and modified until the definitions proposed by the WHO were established created a bias in the field. Diagnosis was initially based on serology and the determination of NS1 (as of 2005), and it was years before the implementation of the clinical-epidemiological criteria. In recent years, antigenic identification with PCR and sequencing to determine the circulating serotypes has been the primary focus. [4,5] Despite this, the epidemiological surveillance system for dengue is robust and capable of identifying changes in patterns, trends, and affected groups, as well as identifying the circulation of serotypes. The introduction of Chikungunya and Zika in 2014 and 2015, respectively, improved this diagnostic method. In this regard, a study conducted in Mexico found an overlap of diseases transmitted by Aedes with 61.7% for Dengue and Zika and 53.3% for Dengue and Chikungunya [40]. Although in our study, only the analysis of Dengue was performed, it is possible that other diseases have a positive correlation with the increase in temperature over the years. Likewise, because this is an ecological study, there may be errors in the results, so it is necessary to continue investigations that facilitate prevention before the inevitable spread of the *Aedes* vector to worldwide and, as a consequence, the increase in Arbovirosis. Although there are various publications on the spread of the vector, there is no historical public atlas (such as the one that was possible to consult the cases and incidence rate of dengue, as well as the climatic variables) in order to be able to carry out a more in-depth analysis. The strengths of the study are that it covered an extensive period and considered important geographical variables and grouped the data into zones and five-year periods.

## 5. Conclusions

In conclusion, in the study, the average temperature increase in Mexico was observed to be 1.6 °C over a period of 38 years. The highest global correlation was with the mean temperature, and the degree of rainfall was not strongly correlated with the dengue incidence rate except in the eastern part of the country, altitude also played a moderating role, with higher altitudes linked to lower incidence rates.

## Figures and Tables

**Table 1:** Temperature correlation with dengue incidence in Mexico 1985 to 2023.

Estimator	Maximum Temperature		Average Temperature		Minimum Temperature		Rainfall	
	CD	HD	CD	HD	CD	HD	CD	HD
b	9.67	3.39	8.03	3.46	7.20	2.13	0.06	0.04
*p* value b	0.25	0.06	0.26	0.01	0.27	0.01	0.69	0.19
R^2^	0.22	0.48	0.20	0.68	0.20	0.68	0.03	0.27
*p*	0.25	0.06	0.26	0.01	0.27	0.01	0.69	0.19
Pearson	0.46	0.69	0.45	0.83	0.45	0.83	0.17	0.52
*p* Pearson	0.25	0.06	0.26	0.01	0.27	0.01	0.69	0.19
Spearman	0.48	0.74	0.40	0.79	0.36	0.71	0.16	0.38
*p* Spearman	0.23	0.04	0.33	0.02	0.39	0.05	0.69	0.35

CD = Classic dengue/non-severe dengue; HD = Dengue haemorrhagic fever/severe dengue/dengue with warning signs.

**Table 2:** Correlation of temperature and rainfall with the incidence of classic dengue in Mexico from 1985 to 2023 according to the region.

Clime	Estimator	Centre-North	Centre-South	Northeast	Northwest	West	East	Southeast	Southwest
Maxim temperture (°C)	b	1.44	0.90	2.74	6.06	−11.82	6.47	−96.43	24.34
	*p* value b	0.02	0.00	0.79	0.47	0.73	0.02	0.06	0.22
	R^2^	0.64	0.83	0.01	0.09	0.02	0.61	0.47	0.23
	*p*	0.02	0.00	0.79	0.47	0.73	0.02	0.06	0.23
	Pearson	0.80	0.91	0.11	0.30	−0.14	0.78	−0.69	0.48
	*p* Pearson	0.02	0.00	0.79	0.47	0.73	0.02	0.06	0.23
	Spearman	0.88	0.71	−0.05	0.38	−0.29	0.64	−0.34	0.54
	*p* Spearman	0.00	0.05	0.91	0.35	0.49	0.09	0.41	0.17
Average temperature (°C)	b	1.56	0.56	−2.45	5.25	10.61	6.43	21.96	19.85
	*p* value b	0.02	0.00	0.81	0.10	0.67	0.00	0.46	0.18
	R^2^	0.60	0.76	0.01	0.39	0.03	0.80	0.01	0.28
	*p*	0.02	0.00	0.81	0.10	0.67	0.00	0.46	0.18
	Pearson	0.78	0.87	−0.10	0.63	0.18	0.89	0.31	0.53
	*p* Pearson	0.02	0.00	0.81	0.10	0.67	0.00	0.46	0.18
	Spearman	0.83	0.75	0.08	0.79	0.24	0.76	0.76	0.54
	*p* Spearman	0.01	0.03	0.84	0.02	0.57	0.03	0.03	0.17
Minimum temperature (°C)	b	1.17	0.34	−3.62	3.46	10.67	5.99	17.48	23.72
	*p* value b	0.15	0.02	0.69	0.10	0.51	0.00	0.19	0.11
	R^2^	0.31	0.61	0.03	0.39	0.07	0.88	0.26	0.36
	*p*	0.15	0.02	0.69	0.10	0.51	0.00	0.19	0.11
	Pearson	0.55	0.78	−0.17	0.62	0.27	0.93	0.51	0.60
	*p* Pearson	0.15	0.02	0.69	0.10	0.51	0.00	0.19	0.11
	Spearman	0.79	0.93	0.12	0.74	0.38	0.76	0.69	0.54
	*p* Spearman	0.02	0.00	0.78	0.04	0.35	0.02	0.06	0.17
Rainfall	b	−0.01	0.00	−0.17	0.04	−0.01	0.02	0.11	−0.05
	*p* value b	0.16	0.39	0.12	0.21	0.87	0.26	0.58	0.34
	R^2^	0.30	0.12	0.36	0.24	0.00	0.20	0.05	0.15
	*p*	0.16	0.39	0.12	0.21	0.88	0.26	0.58	0.34
	Pearson	−0.55	0.35	−0.60	0.49	−0.06	0.45	0.23	−0.39
	*p* Pearson	0.16	0.39	0.12	0.21	0.88	0.26	0.58	0.34
	Spearman	−0.12	0.21	−0.45	0.48	0.10	0.50	0.14	−0.61
	*p* Spearman	0.77	0.61	0.26	0.23	0.82	0.21	0.74	0.10

**Table 3:** Correlation of temperature with the incidence of dengue haemorrhagic disease in Mexico 1985 to 2023 adjusted by rainfall according to the region.

Temperature (°C)	Estimator	Centre-North	Centre-South	Northeast	Northwest	West	East	Southeast	Southwest
Maximum	b_1_	0.02	0.050	0.26	0.80	1.36	0.45	−55.66	9.89
	*p* value b_1_	0.14	0.01	0.34	0.57	0.74	0.00	0.03	0.38
	b_2_	0.00	0.00	0.00	0.00	0.02	0.00	0.02	0.00
	*p* value b_2_	1.00	0.68	0.27	0.40	0.08	0.45	0.71	0.92
	R^2^	0.44	0.82	0.41	0.17	0.49	0.92	0.66	0.27
	*p*	0.24	0.01	0.26	0.64	0.18	0.00	0.07	0.45
Average	b_1_	0.02	0.01	0.17	0.28	4.28	0.37	2.15	8.93
	*p* value b_1_	0.07	0.08	0.51	0.65	0.15	0.00	0.89	0.33
	b_2_	0.00	0.00	−0.01	0.00	0.01	0.00	0.04	0.01
	*p* value b_2_	0.64	0.85	0.22	0.58	0.19	0.54	0.71	0.79
	R^2^	0.56	0.54	0.35	0.15	0.67	0.92	0.03	0.30
	*p*	0.13	0.15	0.34	0.67	0.06	0.00	0.92	0.41
Minimum	b_1_	0.02	0.01	0.16	0.25	3.88	0.34	4.13	15.39
	*p* value b_1_	0.21	0.18	0.49	0.55	0.08	0.00	0.59	0.01
	b_2_	0.00	0.00	0.00	0.00	0.01	0.00	0.03	0.03
	*p* value b_2_	0.40	0.88	0.20	0.66	0.49	0.44	0.73	0.35
	R^2^	0.36	0.38	0.36	0.17	0.74	0.90	0.09	0.53
	*p*	0.33	0.30	0.33	0.62	0.03	0.00	0.79	0.15

**Table 4:** Correlation of temperature and rainfall with the incidence of dengue in Mexico 1985 to 2023 according to the height above sea level (m).

Clime	Estimator	CD				HD			
		<500	500 to 1500	1501 to 2000	>2000	<500	500 to 1500	1501 to 2000	>2000
Maxim temperature	b	4.38	19.63	3.40	0.78	−5.85	5.279	0.06	0.07
	*p* value b	0.85	0.20	0.04	0.41	0.44	0.03	0.06	0.00
	R^2^	0.01	0.26	0.55	0.11	0.10	0.59	0.48	0.80
	*p*	0.85	0.20	0.04	0.41	0.44	0.03	0.06	0.00
	Pearson	0.08	0.51	0.74	0.34	−0.32	0.77	0.70	0.89
	*p* Pearson	0.85	0.20	0.04	0.41	0.44	0.03	0.06	0.00
	Spearman	0.19	0.60	0.85	0.87	0.07	0.83	0.67	0.77
	*p* Spearman	0.65	0.12	0.01	0.00	0.86	0.01	0.07	0.03
Average temperature	b	28.91	14.55	4.43	1.21	6.83	4.02	0.07	0.06
	*p* value b	0.09	0.15	0.01	0.08	0.28	0.01	0.03	0.00
	R^2^	0.40	0.31	0.72	0.43	0.19	0.75	0.55	0.84
	*p*	0.09	0.15	0.01	0.08	0.28	0.01	0.04	0.00
	Pearson	0.63	0.56	0.85	0.66	0.44	0.87	0.74	0.91
	*p* Pearson	0.09	0.15	0.01	0.08	0.28	0.01	0.04	0.00
	Spearman	0.63	0.44	0.85	0.81	0.67	0.86	0.77	0.76
	*p* Spearman	0.10	0.27	0.01	0.01	0.07	0.01	0.03	0.03
Minimum temperature	b	15.57	12.04	3.60	1.22	5.11	2.37	0.05	0.05
	*p* value b	0.08	0.06	0.00	0.05	0.09	0.03	0.02	0.01
	R^2^	0.43	0.47	0.82	0.49	0.40	0.58	0.68	0.75
	*p*	0.08	0.06	0.00	0.05	0.09	0.03	0.02	0.01
	Pearson	0.65	0.68	0.91	0.70	0.63	0.76	0.79	0.86
	*p* Pearson	0.08	0.06	0.00	0.05	0.09	0.03	0.02	0.01
	Spearman	0.48	0.60	0.86	0.81	0.72	0.60	0.76	0.76
	*p* Spearman	0.23	0.12	0.01	0.01	0.04	0.12	0.03	0.03
Rainfall	b	0.04	0.04	−0.01	0.00	0.03	0.01	0.00	0.00
	*p* value b	0.48	0.41	0.52	0.85	0.06	0.03	0.09	0.11
	R^2^	0.08	0.11	0.07	0.00	0.47	0.59	0.40	0.37
	*p*	0.49	0.41	0.52	0.85	0.06	0.03	0.09	0.11
	Pearson	0.29	0.34	−0.27	−0.08	0.68	0.77	−0.63	−0.61
	*p* Pearson	0.49	0.41	0.5171	0.86	0.06	0.03	0.09	0.11
	Spearman	0.38	0.31	−0.24	−0.10	0.49	0.62	−0.55	−0.33
	*p* Spearman	0.35	0.46	0.57	0.88	0.22	0.10	0.16	0.43

CD = Classic dengue/non-severe dengue. HD = Dengue haemorrhagic fever/severe dengue/dengue with warning signs.

**Table 5:** Correlation of temperature and rainfall with the incidence of dengue in Mexico 1985 to 2023 according to the region.

Clime	Estimator	Centre-North	Centre-South	Northeast	Northwest	West	East	Southeast	Southwest
Maximun temperature	b	0.02	0.02	0.33	0.52	0.09	0.48	−56.30	9.09
	*p* value b	0.07	0.00	0.22	0.70	0.99	0.00	0.01	0.19
	R^2^	0.44	0.81	0.23	0.03	0.00	0.91	0.65	0.27
	*p*	0.07	0.00	0.22	0.70	0.99	0.00	0.01	0.19
	Pearson	0.66	0.90	0.48	0.16	0.01	0.96	−0.81	0.52
	*p* Pearson	0.07	0.00	0.22	0.70	0.99	0.00	0.01	0.19
	Spearman	0.74	0.77	0.41	0.53	0.37	0.90	−0.23	0.65
	*p* Spearman	0.04	0.02	0.31	0.17	0.37	0.00	0.58	0.08
Average temperature	b	0.03	0.03	0.21	0.38	6.18	0.42	2.25	7.06
	*p* value b	0.04	0.04	0.44	0.48	0.04	0.00	0.88	0.17
	R^2^	0.54	0.53	0.10	0.09	0.52	0.91	0.00	0.29
	*p*	0.04	0.04	0.44	0.48	0.04	0.00	0.88	0.17
	Pearson	0.73	0.73	0.32	0.29	0.72	0.95	0.06	0.54
	*p* Pearson	0.04	0.04	0.44	0.48	0.04	0.00	0.88	0.17
	Spearman	0.73	0.77	0.43	0.84	0.87	0.88	0.79	0.65
	*p* Spearman	0.04	0.03	0.2830	0.01	0.00	0.00	0.02	0.08
Minimum temperatura	b	0.02	0.015	0.165	0.32	4.75	0.36	4.330	8.97
	*p* value b	0.20	0.11	0.51	0.37	0.01	0.00	0.54	0.08
	R^2^	0.25	0.38	0.08	0.14	0.71	0.89	0.07	0.43
	*p*	0.20	0.11	0.51	0.37	0.01	0.00	0.54	0.08
	Pearson	0.50	0.61	0.28	0.37	0.84	0.94	0.26	0.66
	*p* Pearson	0.20	0.11	0.51	0.37	0.01	0.00	0.54	0.08
	Spearman	0.49	0.77	0.27	0.84	0.74	0.88	0.83	0.67
	*p* Spearman	0.2220	0.03	0.51	0.01	0.03	0.00	0.01	0.07
Rainfall	b	0.00	0.00	−0.01	0.00	0.02	−0.01	0.04	−0.02
	*p* value b	0.47	0.52	0.17	0.43	0.06	0.01	0.68	0.36
	R^2^	0.09	0.07	0.29	0.11	0.48	0.39	0.03	0.14
	*p*	0.47	0.52	0.17	0.43	0.06	0.01	0.68	0.36
	Pearson	−0.30	0.27	−0.53	0.33	0.69	0.62	0.17	0.37
	*p* Pearson	0.47	0.52	0.17	0.43	0.06	0.70	0.68	0.36
	Spearman	−0.31	0.48	−0.20	0.29	0.68	0.46	0.32	−0.59
	*p* Spearman	0.45	0.23	0.63	0.48	0.06	0.25	0.43	0.13

**Table 6:** Temperature correlation with dengue incidence in Mexico 1985 to 2023 adjusted by rainfall according to height above sea level (m).

Temperature	Estimator	CD				HD			
		<500	500 to 1500	1501 to 2000	>2000	<500	500 to 1500	1501 to 2000	>2000
Maximum	b_1_	10.42	18.66	3.59	1.17	−2.06	3.27	0.042	0.07
	*p* value b_1_	0.68	0.37	0.06	0.39	0.75	0.17	0.16	0.02
	b_2_	0.05	0.01	0.00	0.00	0.03	0.10	0.00	0.00
	*p* value b_2_	0.48	0.94	0.79	0.65	0.12	0.17	0.27	0.91
	R^2^	0.12	0.26	0.56	0.15	0.48	0.73	0.60	0.80
	*p*	0.73	0.48	0.13	0.66	0.20	0.04	0.10	0.02
Average	b_1_	29.35	22.36	4.42	1.38	2.25	3.38	0.06	0.05
	*p* value b_1_	0.17	0.24	0.02	0.09	0.71	0.11	0.06	0.00
	b_2_	0.00	−0.04	0.00	0.00	0.03	0.00	0.00	0.00
	*p* value b_2_	0.96	0.59	0.99	0.56	0.15	0.68	0.14	0.12
	R^2^	0.40	0.35	0.72	0.47	0.48	0.76	0.72	0.90
	*p*	0.28	0.34	0.04	0.20	0.19	0.03	0.04	0.00
Minimum	b_1_	16.70	20.52	3.597	1.31	3.00	1.28	0.05	0.05
	*p* value b_1_	0.14	0.06	0.01	0.07	0.34	0.40	0.06	0.01
	b_2_	−0.01	−0.07	0.00	0.00	0.02	0.01	0.00	0.00
	*p* value b_2_	0.83	0.27	0.98	0.64	0.22	0.37	0.13	0.08
	R^2^	0.43	0.59	0.82	0.51	0.56	0.65	0.72	0.87
	*p*	0.24	0.11	0.01	0.17	0.12	0.07	0.04	0.01

CD = Classic dengue/non-severe dengue. HD = Dengue haemorrhagic fever/severe dengue/dengue with warning signs.

## Data Availability

DOI: 10.6084/m9.figshare.28054115.

## Supplementary Materials

The following supporting information can be downloaded at: https://www.mdpi.com/article/10.3390/microorganisms12122661/s1, Table S1: Average temperature and rainfall per five-year period from 1985 to 2023 and region; Table S2: Average dengue incidence rate per five-year period from 1985 to 2023 and region; Table S3: Average temperature and rainfall per five-year period from 1985 to 2023 and height above sea level (m); Table S4: Average dengue incidence rate per five-year period from 1985 to 2023 and height above sea (m).

## References

[1] Espinoza-Gomez F., Newton-Sanchez O.A., Nava-Zavala A.H., Zavala-Cerna M.G., Rojas-Larios F., Delgado-Enciso I., Martinez-Rizo A.B., Lozano-Kasten F. (2020). Demographic and climatic factors associated with dengue prevalence in a hyperendemic zone in Mexico: An empirical approach. Trans. R. Soc. Trop. Med. Hyg..

[2] Gaspar-Castillo C., Cortes-Escamilla A., Aparicio-Antonio R., Carnalla M., López S., Sánchez-Tacuba L., Oceguera-Cabrera A., Burrone Ó., González-Bonilla C., Navarrete V.O. (2024). Evolution of Zika prevalence in a dengue hyperendemic municipality in Southern Mexico after the outbreak of 2015 to 2017. Salud Publica Mex..

[3] Dirección General de Epidemiología Panorama Epidemiológico de Dengue. https://www.gob.mx/salud/acciones-y-programas/informacion-epidemiologica.

[4] Kuri-Morales P., Correa-Morales F., González-Acosta C., Sánchez-Tejeda G., Dávalos-Becerril E., Juárez-Franco M.F., Díaz-Quiñonez A., Huerta-Jimenéz H., Mejía-Guevara M.D., Moreno-García M. (2017). First report of *Stegomyia aegypti* (=*Aedes aegypti*) in Mexico City, Mexico. Med. Vet. Entomol..

[5] Dávalos-Becerril E., Correa-Morales F., González-Acosta C., Santos-Luna R., Peralta-Rodríguez J., Pérez-Rentería C., Ordoñez-Álvarez J., Huerta H., Carmona-Perez M., Díaz-Quiñonez J.A. (2019). Urban and semi-urban mosquitoes of Mexico City: A risk for endemic mosquito-borne disease transmission. PLoS ONE.

[6] Brady O.J., Golding N., Pigott D.M., Kraemer M.U.G., Messina J.P., Reiner R.C., Scott T.W., Smith D.L., Gething P.W., Hay S.I. (2014). Global temperature constraints on *Aedes aegypti* and *Ae. albopictus* persistence and competence for dengue virus transmission. Parasites Vectors.

[7] Gwee X.W.S., Chua P.E.Y., Pang J. (2021). Global dengue importation: A systematic review. BMC Infect. Dis..

[8] Hii Y.L., Zhu H., Ng N., Ng L.C., Rocklöv J. (2012). Forecast of Dengue Incidence Using Temperature and Rainfall. PLoS Neglected Trop. Dis..

[9] Shil P. (2019). Rainfall and Dengue Occurrences in India during 2010–2016. Biomed. Res. J..

[10] Ehelepola N.D.B., Ariyaratne K., Buddhadasa W.M.N.P., Ratnayake S., Wickramasinghe M. (2015). A study of the correlation between dengue and weather in Kandy City, Sri Lanka (2003–2012) and lessons learned. Infect. Dis. Poverty.

[11] Hurtado-Díaz M., Riojas-Rodríguez H., Rothenberg S.J., Gomez-Dantés H., Cifuentes E. (2007). Short communication: Impact of climate variability on the incidence of dengue in Mexico. Trop. Med. Int. Health.

[12] Díaz-Castro S., Moreno-Legorreta M., Ortega-Rubio A., Serrano-Pinto V. (2017). Relation between dengue and climate trends in the Northwest of Mexico. Trop. Biomed..

[13] Hernández-Gaytán S.I., Díaz-Vásquez F.J., Duran-Arenas L.G., Cervantes M.L., Rothenberg S.J. (2017). 20 Years Spatial-Temporal Analysis of Dengue Fever and Hemorrhagic Fever in Mexico. Arch. Med. Res..

[14] Servicio Meteorológico Nacional Resúmenes Mensuales de Temperatura y Lluvia. https://smn.conagua.gob.mx/es/climatologia/temperaturas-y-lluvias/resumenes-mensuales-de-temperaturas-y-lluvias.

[15] Mendoza-Cano O., Trujillo X., Huerta M., Ríos-Silva M., Lugo-Radillo A., Benites-Godínez V., Bricio-Barrios J.A., Ríos-Bracamontes E.F., Uribe-Ramos J.M., Baltazar-Rodríguez G.M. (2023). Assessing the Relationship between Annual Surface Temperature Changes and the Burden of Dengue: Implications for Climate Change and Global Health Outcomes. Trop. Med. Infect. Dis..

[16] Jia P., Chen X., Chen J., Lu L., Liu Q., Tan X. (2017). How does the dengue vector mosquito *Aedes albopictus* respond to global warming?. Parasites Vectors.

[17] Gubler D.J., Reiter P., Ebi K.L., Yap W., Nasci R., Patz J.A. (2001). Climate variability and change in the United States: Potential impacts on vector- and rodent-borne diseases. Environ. Health Perspect..

[18] Jansen C.C., Beebe N.W. (2010). The dengue vector *Aedes aegypti*: What comes next. Microbes Infect..

[19] Li Y., Kamara F., Zhou G., Puthiyakunnon S., Li C., Liu Y., Zhou Y., Yao L., Yan G., Chen X.-G. (2014). Urbanization increases *Aedes albopictus* larval habitats and accelerates mosquito development and survivorship. PLoS Neglected Trop. Dis..

[20] Narro-Robles J., Gómez-Dantés H. (1995). El dengue en Mexico: Un problema prioritario de salud pública. Salud Pública Mex..

[21] Vázquez-Castellanos J.L., Canales-Muñoz J.L., Nápoles-Camacho M.A., Castillo-Morán M.A., Ureña-Carillo L.E. (2018). Estudio del primer gran brote epidémico de dengue en Guadalajara, Jalisco, México, octubre de 1988. Rev. Salud Jalisco.

[22] Bautista P.F.H., Gaytán D.A.C., Tinoco C.E.S., Parás A.V., Yaah J.E.A., Miguel B.M., Hernández Y.M.A., Nieto L.A., Paz A.M., Betancourt L.J. (2024). Retrospective Analysis of Severe Dengue by Dengue Virus Serotypes in a Population with Social Security, Mexico 2023. Viruses.

[23] San Martín J.L., Brathwaite O., Zambrano B., Solórzano J.O., Bouckenooghe A., Dayan G.H., Guzmán M.G. (2010). The epidemiology of dengue in the Americas over the last three decades: A worrisome reality. Am. J. Trop. Med. Hyg..

[24] Brathwaite Dick O., San Martín J.L., Montoya R.H., del Diego J., Zambrano B., Dayan G.H. (2012). The history of dengue outbreaks in the Americas. Am. J. Trop. Med. Hyg..

[25] Navarrete J., Vásquez J.L., Vásquez J.A., Gómez H. (2002). Epidemiologia del dengue y dengue hemorrágico en el Instituto Mexicano del Seguro Social (IMSS). Rev. Peruana. Epidemiol..

[26] Instituto Nacional de Estadística y Geografía Agua Potable y Drenaje. https://cuentame.inegi.org.mx/territorio/agua/dispon.aspx?tema=T.

[27] Instituto Nacional de Estadística y Geografía Estadísticas a Propósito del Día Mundial del Agua. 17 de marzo de 2023. https://www.inegi.org.mx/app/saladeprensa/noticia.html?id=8053.

[28] Consejo Nacional de Evaluación de la Política del Desarrollo Social Medición de la pobreza en México. https://www.coneval.org.mx/Medicion/Paginas/PobrezaInicio.aspx.

[29] Torres-Galicia L., Cortés-Poza D., Becker I. (2014). Dengue in Mexico: An analysis of two decades. Gac. Med. Mex..

[30] Ibáñez-Bernal S., Gómez-Dantés H. (1995). Los vectores del dengue en México: Una revisión crítica [Vectors of dengue in Mexico: A critical review)]. Salud Publica Mex..

[31] Ortega-Morales A.I., Pérez-Rentería C., Ordóñez-Álvarez J., Salazar J.A., Dzul-Manzanilla F., Correa-Morales F., Huerta-Jiménez H. (2022). Update on the Dispersal of *Aedes albopictus* in Mexico: 1988–2021. Front. Trop. Dis..

[32] Arredondo-Jiménez J.I., Valdez-Delgado K.M. (2006). *Aedes aegypti* pupal/demographic surveys in southern Mexico: Consistency and practicality. Ann. Trop. Med. Parasitol..

[33] Manrique-Saide P., Coleman P., McCall P.J., Lenhart A., Vázquez-Prokopec G., Davies C.R. (2014). Multi-scale analysis of the associations among egg, larval and pupal surveys and the presence and abundance of adult female *Aedes aegypti* (*Stegomyia aegypti*) in the city of Merida, Mexico. Med. Vet. Entomol..

[34] Lozano-Fuentes S., Hayden M.H., Welsh-Rodriguez C., Ochoa-Martinez C., Tapia-Santos B., Kobylinski K.C., Uejio C.K., Zielinski-Gutierrez E., Monache L.D., Monaghan A.J. (2012). El mosquito vector del virus del dengue *Aedes aegypti* en las grandes alturas de México. Am. J. Trop. Med. Hyg..

[35] Marina C.F., Bond J.G., Hernández-Arriaga K., Valle J., Ulloa A., Fernández-Salas I., Carvalho D.O., Bourtzis K., Dor A., Williams T. (2021). Population Dynamics of *Aedes aegypti* and *Aedes albopictus* in Two Rural Villages in Southern Mexico: Baseline Data for an Evaluation of the Sterile Insect Technique. Insects.

[36] Wiwanitkit V. (2006). An observation on correlation between rainfall and the prevalence of clinical cases of dengue in Thailand. J. Vector Borne Dis..

[37] Gastelbondo-Pastrana B., Echeverri-De la Hoz D., Sanchez L., García Y., Espitia-Delgado Y., Lopez Y., Yasnot-Acosta M.F., Arrieta G., Mattar S. (2024). Climatic variables and their relationship with vector-borne disease cases in Colombia, 2011–2021. Front. Trop. Dis..

[38] Gage K.L., Burkot T.R., Eisen R.J., Hayes E.B. (2008). Climate and vector-borne diseases. Am. J. Prev. Med..

[39] Hales S., de Wet N., Maindonald J., Woodward A. (2002). Potential effects of global climate change on human health: An empirical review. Lancet.

[40] Dzul-Manzanilla F., Correa-Morales F., Che-Mendoza A., Palacio-Vargas J., Sánchez-Tejeda G., González-Roldan J.F., López-Gatell H., E Flores-Suárez A., Gómez-Dantes H., E Coelho G. (2021). Identifying urban hotspots of dengue, chikungunya, and Zika transmission in Mexico to support risk stratification efforts: A spatial analysis. Lancet Planet. Health.

